# Body position influence on cerebrospinal fluid volume redistribution inside the cranial and spinal CSF compartments

**DOI:** 10.3389/fnhum.2024.1463740

**Published:** 2025-01-29

**Authors:** I. Strbačko, M. Radoš, I. Jurjević, D. Orešković, M. Klarica

**Affiliations:** ^1^Croatian Institute for Brain Research, School of Medicine, University of Zagreb, Zagreb, Croatia; ^2^Department of Neurology, University Hospital Centre Zagreb, Zagreb, Croatia; ^3^Department of Molecular Biology, Ruđer Bošković Institute, Zagreb, Croatia; ^4^Department of Pharmacology, School of Medicine, University of Zagreb, Zagreb, Croatia

**Keywords:** cranial CSF volume, spinal CSF volume, body position, volume redistribution, segmentation, MR volumetry

## Abstract

**Introduction:**

It is generally accepted that during body position changes from horizontal to vertical there is a short-lasting shift of a certain CSF volume from the cranium into the hydrostatically lower parts of the spinal space, which leads to transitory CSF pressure decrease to negative values.

**Methods:**

In order to test this, we performed MRI volumetry of cranial and spinal part of the CSF space in healthy volunteers of both genders (*n* = 22) in three different body positions [horizontal (H); elevated head and upper body (H-UP) under an angle about 30° from the base; elevated lower body (B-UP) under an angle about 30° from the base].

**Results:**

Volumes of brain and spinal cord tissue did not change during body position changes. Significant CSF volume (ml) changes occur inside the spinal space in the tested body positions, primarily in the lumbosacral segment (H-UP – 38.1 ± 7.0; H – 34.4 ± 6.5; B-UP – 28.7 ± 6.5), while at the same time no significant CSF volume changes have been observed inside the cranium in two tested positions (H and B-UP) in which it was possible to measure intracranial CSF volume changes or if we sum up cervical and cranial CSF volumes in those positions.

**Conclusion:**

Observed results suggest that during the changes of body position CSF volume redistribution occurs, primarily inside the spinal and not the cranial space. This is in accordance with the new hypothesis by which spinal intradural space can significantly change its volume due to its elasticity, thus adjusting to the influence of gravity and pressure changes.

## Introduction

The intracranial space contains certain volumes of cerebrospinal fluid (CSF), cerebral blood and brain parenchyma. According to Monroe – Kellie doctrine, the sum of those volumes is constant. Thus, this doctrine has theoretical implications in the regulation of CSF pressure and volume (Davson et al., 1987). A classical concept of CSF physiology suggests that CSF flows unidirectionally from the site of its secretion to the site of its absorption (Davson et al., 1987), which implies the existence of pressure gradient inside the CSF system with the highest pressure intracranially at the secretion site and the lowest pressure at the absorption site (Cutler et al., 1968; Pollay, 2010) in order to biophysically enable the supposed unidirectional movement. It is known that during head-up verticalization the intracranial pressure diminishes (Bradley, 1970; Magnæs, 1976a,b; Chapman et al., 1990; Antes et al., 2016), which is considered to be a transitory phenomenon. Namely, the CSF pressure should always stay positive due to continuous secretion, and its temporary decline was explained (according to Monroe-Kellie doctrine) in light of a potential short-term CSF volume redistribution from the cranial into the spinal part of the CSF system under the influence of gravity (Magnaes, 1978; Davson et al., 1987; Magnæs, 1989).

Novel research about the influence of body position on the intracranial pressure values in patients is also interpreted by a classical concept of CSF pressure regulation, according to which CSF pressure depends on the rate of CSF secretion (Vf), the resistance of the CSF circulation pathway (Ro) and the value of venous pressure (Pv) inside the dural sinuses [CSF pressure = (Vf × Ro) + Pv] where CSF absorption hypothetically occurs (Alperin et al., 2005a,b, 2016; Qvarlander et al., 2013; Holmlund et al., 2018; Linden et al., 2018). It is believed that a decrease of venous pressure inside the cranium during body verticalization diminishes CSF circulation resistance and facilitates CSF absorption into the dural sinuses (Qvarlander et al., 2013; Holmlund et al., 2018; Linden et al., 2018), suggesting that in this position a transitory decrease of intracranial CSF volume occurs.

About 15 years ago a novel concept regarding the physiology and pathophysiology of fluids inside the craniospinal space was created, according to which CSF, interstitial fluid and blood are interconnected, and the net water turnover between those three compartments depends on the gradients of hydrostatic and osmotic forces that are present between the central nervous system tissue capillaries, interstitial fluid and CSF (Bulat – Klarica –Orešković hypothesis) (Orešković and Klarica, 2010, 2011; Bulat and Klarica, 2011; Naidich et al., 2013; Thomale, 2021; Atchley et al., 2022; Theologou et al., 2022; Bajda et al., 2023). Thus, according to this concept, the CSF pressure is not dependent on the velocity of secretion inside the ventricles, the resistance to unidirectional CSF circulation from the ventricles to the cortical subarachnoid space or the pressure inside the venous sinuses (the dominant site of absorption) and that clearance of brain metabolites takes place locally (near to the site of their production) through various transport systems of the capillary network.

The role of respiration and blood vessels pulsation in CSF dynamics is being extensively researched (Balendet et al., 2004; Yamada, 2014; Orešković and Klarica, 2014), and it was observed that pulsatile CSF to-and-fro movements significantly change in various pathophysiological conditions. During the last 12 years, a glymphatic pathway concept has been developed and described in the literature (Iliff et al., 2012, 2013; Nedergaard and Goldman, 2020; Bohr et al., 2022; Smets et al., 2023). It’s believed that movement of different marker substances via perivascular space into and out of the brain tissue following their application into the CSF system can help the clearance of brain metabolic waste (probably important for some neurodegenerative diseases) (Iliff et al., 2012; Nedergaard and Goldman, 2020).

Examinations done on larger experimental animals about 10 years ago (Klarica et al., 2014) implied that the intracranial CSF pressure is continuously negative during the vertical head-up position and that this is not a transitory phenomenon but a physiological CSF state inside the cranium. If the intracranial CSF pressure is continuously negative and stable in the upright position and if this is not a transitory observation, a question arises as to what is happening with the CSF volume? Is the CSF volume really redistributed between the cranial and the spinal CSF space under the influence of gravity and does this potential volume change also affect the CSF pressure value and compliance alteration in individual CSF compartments? In order to answer these questions and to improve the understanding of CSF physiology as well as CSF volume and pressure regulation in individual segments of the CSF system, we analyzed CSF volumes inside the cranial and spinal CSF space in three different body positions in healthy volunteers.

## Materials and methods

### Research participants

This research was done on 22 healthy volunteers, of which 11 were male and 11 were female. Subjects were 20 to 34 years old, 25.5 years on average. Their height varied from 158 to 187 cm, while they weighed between 50 and 110 kg (see Supplementary Table S1). Before the MRI imaging, all subjects filled out a standardized questionnaire where they stated their brief medical history, if they had any severe diseases or surgical procedures. We, as medical doctors, reviewed each form to ensure that our subjects were healthy (no significant health problems) and had not had surgery that could affect the cerebrospinal system. Additional analysis was done based on performed MRI imaging, and any pathological condition inside the craniospinal system was excluded.

This research gained a positive opinion of the Ethics Committee of the University of Zagreb School of Medicine (Reg. number: 380-59-10106-15-168/39, Class: 641-01/15-02/01). All subjects were thoroughly acquainted with the method of magnetic resonance imaging in writing and orally, and filled out a questionnaire with their basic and medical data in order to determine whether there were any contraindications to the safety of MRI imaging. All data obtained and used during this research are protected, anonymized and stored in the appropriate database.

### MRI volumetry

**Figure 1:**
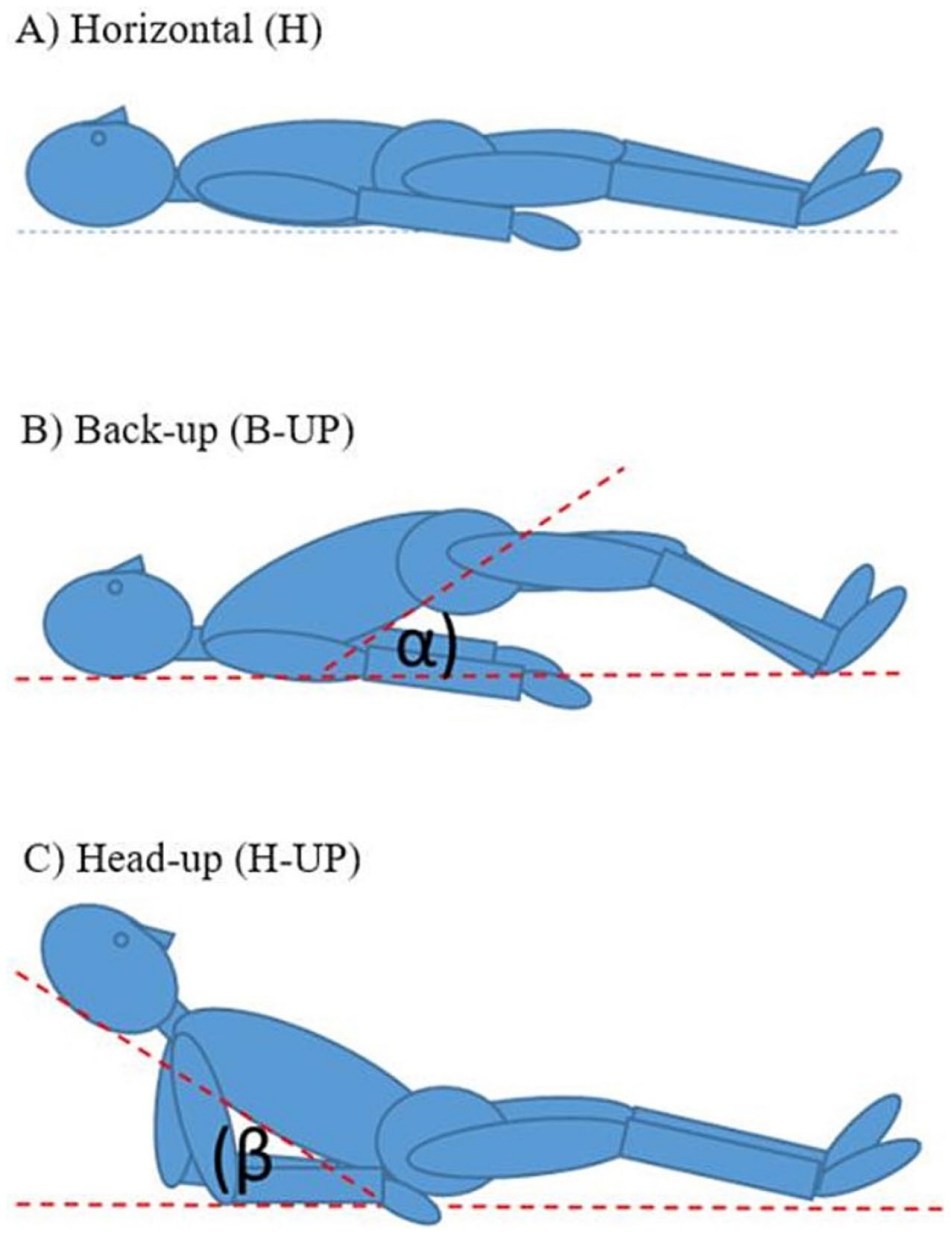
Schematic representation of the body position during MRI imaging: **(A)** horizontal position (H), **(B)** elevated lower body position (B-UP) under the certain angle *α* (about 30°) in relation to the base, **(C)** elevated head and the upper body position (H-UP) under the certain angle *β* (about 30°) in relation to the base.

MRI imaging was done in Polyclinic „Neuron” situated in Croatian Institute for Brain Research, on the MRI device with a 3 T magnetic field (Magnetom Prisma^FIT^, Siemens, Germany). The width of the MRI device tunnel is standard and measures 60 cm. For cranial imaging, high-resolution sagittal MPRAGE T1 sequences were used, and spinal imaging was performed using high—resolution sagittal T2 sequences. Subjects were recorded in three different body positions: (1) horizontal position (H); (2) elevated lower body position (B-UP) under a certain angle *α* (about 30°) from the base; and (3) elevated head and upper body position (H-UP) under a certain angle *β* (about 30°) from the base (Figure 1).

For cranial imaging we used a 64-canal head and neck coil which is fixed to the MRI device bed and cannot be moved, so each participant underwent cranial imaging in two positions H and B-UP. The spinal part of the system was recorded in all three positions in each subject – for spinal imaging we used the coil fixed in the MRI device bed and a large flexible coil (Big Flexi) for spinal imaging in the B-UP position. The duration of MRI imaging per subject in all three positions was from 46 min to a maximum of 1 h and 18 min, with the average duration of 59 min and 57 s. Imaging of the cranial and spinal parts in the horizontal position (H) lasted from 17 to 36 min, an average of 23 min. Imaging of the cranial and spinal part in the elevated lower body position (B-UP) lasted from 16 to 41 min, about 24 min and 3 s on average. Imaging in the elevated head and upper body position (H-UP) was the shortest since only the spinal part was recorded, and lasted from 9 to a maximum of 20 min, an average of 12 min and 55 s.

High-resolution sagittal T1 sequences (TR/TE = 2300/3 ms, FOV = 250 × 250, voxel dimensions: 0.97 × 0.97 × 1 mm) were used for the cranial part, which are suitable for precise morphometric analysis and for automatic segmentation of the cranium. For the spinal part, high-resolution sagittal T2 sequences were used (for the horizontal position TR/TE = 1700/221 ms, FOV = 340 × 340 mm, voxel dimensions = 1.08 × 1.06 × 1.06 mm; for the position with the head and upper body raised for cervical and thoracic part TR/TE = 1700/224 ms, FOV = 390 × 390 mm, voxel dimensions 1.24 × 1.21 × 1.22 mm; for the position with the head and upper body raised for lumbosacral part TR/TE = 1980/224 ms, FOV = 380 × 380 mm, voxel dimensions: 1.21 × 1.18 × 1.25 mm; for the position with the lower body raised for cervical and thoracic part TR/TE = 1700/224 ms, FOV = 390 × 390 mm, voxel size: 1.24 × 1.21 × 1.22 mm; for position with the lower body raised for lumbosacral part TR = 1980/224 ms, FOV = 380 × 380 mm, voxel size: 1.21 × 1.18 × 1.25 mm) which provide a good contrast between the cerebrospinal fluid and the surrounding tissue, i.e., enable clear detection of the edges of the cerebrospinal fluid space, which is necessary for high-quality volumetric analysis and segmentation.

### Volumetric analysis

**Figure 2:**
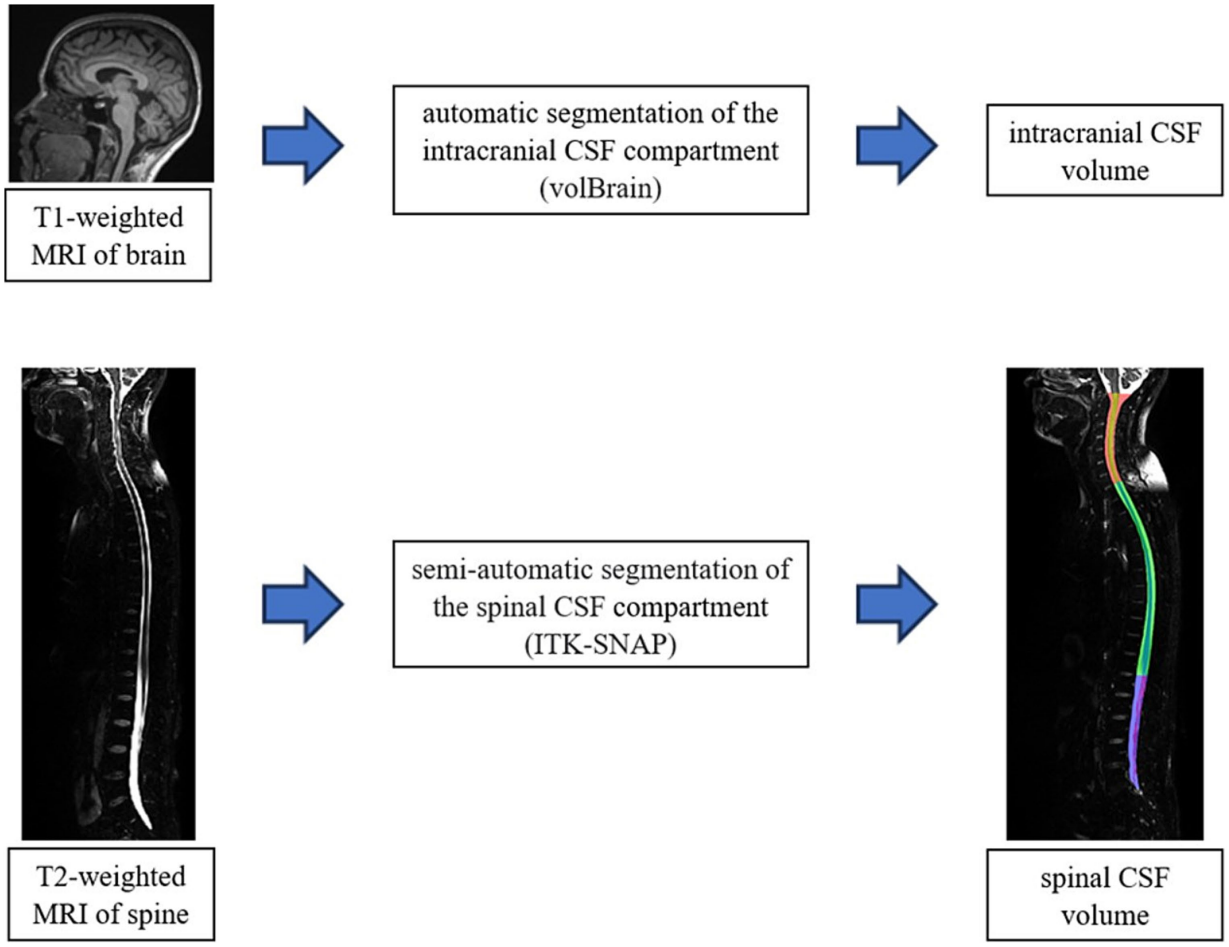
Schematic representation of the volumetric analysis process of MRI images of the brain (volBrain) and spine (ITK-SNAP). The final result of the segmentation of the spinal part (cervical-red, thoracic-green and lumbosacral-blue part) in the ITK-SNAP programme is shown in the down part of scheme.

For the interpretation of intracranial MRI imaging, we used a verified *online* programme for brain imaging analysis volBrain (Manjón and Coupé, 2016) (Figure 2). This programme was used for a quantitative analysis of the MRI signal intensity, and an automated segmentation was used to determine the total brain volume, as well as specific volumes of grey and white matter, the total CSF volume intracranially and the volume of lateral ventricles.

Due to the atypical positions of the subjects during the recording, we could not use automatic segmentation for the spinal part. For the spinal part analysis, we used a semi-automated segmentation method with the ITK SNAP programme (Yushkevich et al., 2006) (Figure 2) which, even though it is time-consuming and requires manual volume tracing, enables obtaining precise dana which was very significant considering atypical positions of the participants inside the MRI device. We analyzed the volumes of the total spinal compartment, as well as cervical, thoracic and lumbosacral parts separately, including the volumes of the spinal medulla and the cerebrospinal fluid in all three body positions.

### Statistical analysis

We used Kolmogorov–Smirnov test to analyze the distribution of all continuous values, and the differences between the groups were analyzed by one-way variance analysis (engl. One Way ANOVA) with an additional *post-hoc* Bonferroni test in the case of significant differences. Graphic displays of the differences in continuous values showed the arithmetic mean with concomitant 95% confidence intervals. All *p* values under 0.05 were considered statistically significant. A licensed programme support MedCalc^®^ Statistical Software version 20.106 (MedCalc Software Ltd., Ostend, Belgium; https://www.medcalc.org; 2022) was used for the analysis.

## Results

### Volumetric analysis of the brain and spinal medulla MRI images in different body positions

Since it is normally expected that brain and spinal medulla volumes do not change during the changes of body position, a comparation of these volumes is significant as a kind of control method for both automated brain segmentation and semiautomated medulla segmentation. By analyzing brain volumes and volumes of medulla spinalis in total as well as each (cervical, thoracic and lumbosacral) segment individually, it was determined that there was no statistically significant difference between those volumes in different body positions, which implies that our measuring methods were reliable and our results plausible. The average brain volume in the H position was 1330.4 ± 112.1 mL; of which white mater volume was averagely 556.7 ± 66.7 mL; while the average grey matter volume was 773.8 ± 62.9 mL. In the B-UP position, the average brain volume amounted to 1333.3 ± 107.4 mL; the average white matter volume was 553.6 ± 63.5 mL; while the average grey matter volume was 779.8 ± 58.9 mL. The average measured value of the total spinal medulla volume was 29.8 ± 3.5 mL in the H position, 29.4 ± 3.1 mL in the B-UP position and 30.5 ± 3.2 mL in the H-UP position.

### Intracranial CSF volume

Using automated segmentation in the volBrain programme, volumes of total intracranial CSF and of lateral ventricles CSF were measured in two positions: H position and B-UP position. By subtraction of the lateral ventricles CSF volume from the total intracranial CSF volume, the approximate value of subarachnoid CSF volume was obtained (taking into account that this volume also contains the volumes of the third and the fourth ventricle which were not individually analyzed in the volBrain programme).

**Figure 3:**
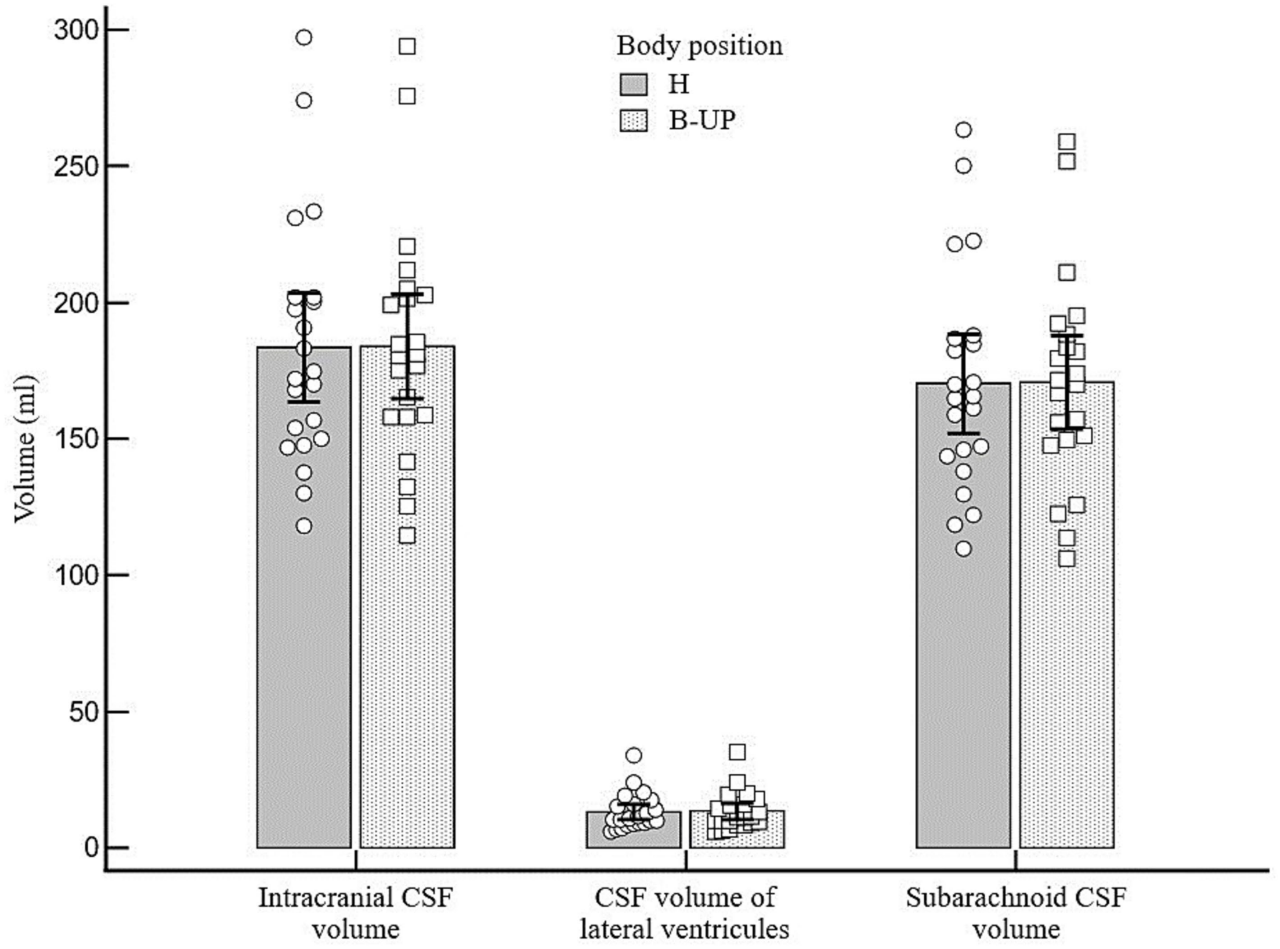
Differences between the measured values of intracranial, ventricular and subarachnoid CSF volumes (ml) related to different body positions during MRI imaging. Columns represent the values of arithmetic means of intracranial, lateral ventricles and subarachnoid CSF volumes with their 95% confidence intervals in the horizontal position (H) and in the position with the elevated lower body (B-UP), while white circles and squares represent individual values of those volumes in 22 subjects. There were no statistically significant differences in intracranial, ventricular and cranial subarachnoid CSF volumes in different body positions.

By analyzing the obtained results, it was determined that there is no statistically significant difference in the volumes of the total intracranial CSF, lateral ventricles CSF or subarachnoid CSF between two studied body positions in which there were no changes of the head position (H and B-UP) (Figure 3). The average intracranial CSF volume value in H position amounts to 183.5 ± 44.9 mL, the average volume of lateral ventricles CSF was 13.2 ± 6.6 mL, and the average subarachnoid CSF volume was 170.2 ± 40.7 mL. In the B-UP position, the average intracranial CSF volume value was 184.0 ± 43.2 mL, average CSF volume of lateral ventricles was 13.4 ± 6.8 mL, and the average subarachnoid CSF volume was 170.6 ± 38.6 mL.

### Influence of body position on the spinal CSF volume distribution

**Figure 4:**
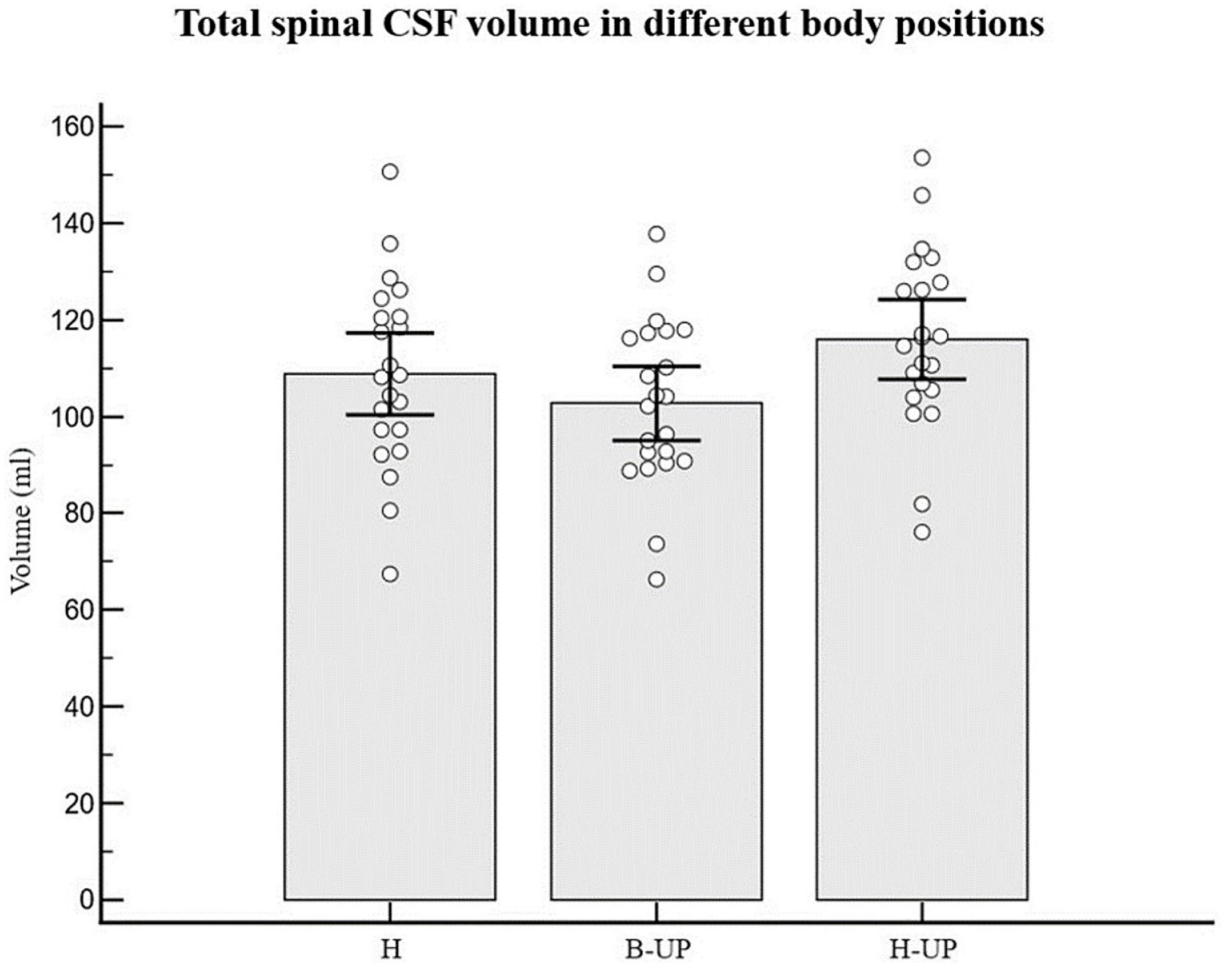
Differences between measured values of the total spinal CSF volume (ml) related to body position during MRI imaging. Columns represent the values of arithmetic means of total spinal CSF volume with their 95% confidence intervals in horizontal position (H), in the position with elevated lower body (B-UP) and in the position with elevated head and upper body (H-UP), while white circles represent individual values of those volumes in 22 subjects. There were no statistically significant differences between the measured total spinal CSF volume in different body positions.

Analyzing the values of the total spinal CSF volume in 22 subjects and in three different body positions (H, B-UP and H-UP), it was determined that there was no statistically significant difference (Figure 4). The average value of the spinal CSF volume was 108.8 ± 19.2 mL in the H position, 102.8 ± 17.3 mL in the B-UP position and 115.9 ± 18.5 mL in the H-UP position. However, it is our observation that there is a tendency to volume change, especially during head lift. In addition, we analyzed individual values of the CSF volumes in the cervical and thoracic vertebral segments in 22 subjects in three different body positions, and it was also determined that there was no statistically significant difference, even when we summed up the cervical and thoracic segment and compared them that way.

### Influence of body position on the lumbosacral CSF volume distribution

**Figure 5:**
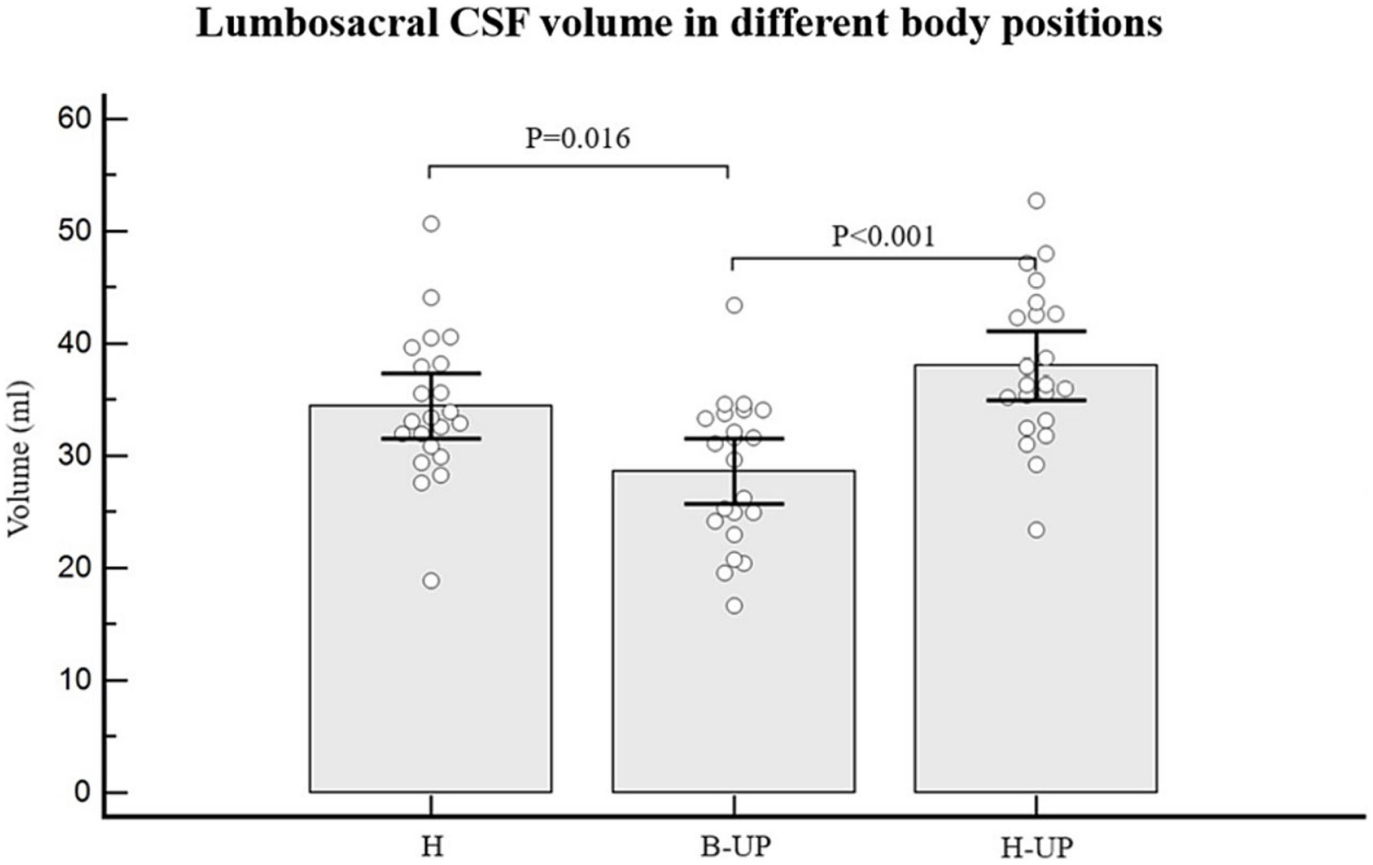
Differences between measured values of lumbosacral CSF volume (ml) related to the changes of body position during MRI imaging. Columns represent the values of arithmetic means of lumbosacral CSF volumes with their 95% confidence intervals in the horizontal position (H), in the position with the elevated lower body (B-UP) and in the position with the elevated head and upper body (H-UP), while white circles represent individual values of those volumes in 22 subjects. The analysis of variance determined significant difference between the three MRI measuring positions (*p* < 0.001). An additional post-hoc Bonferroni analysis showed significant differences primarily between the horizontal (H) position and the elevated lower body position (B-UP) (*p* = 0.016), as well as between the elevated head and upper body position (H-UP) and the elevated lower body position (B-UP) (*p* < 0.001).

**Figure 6:**
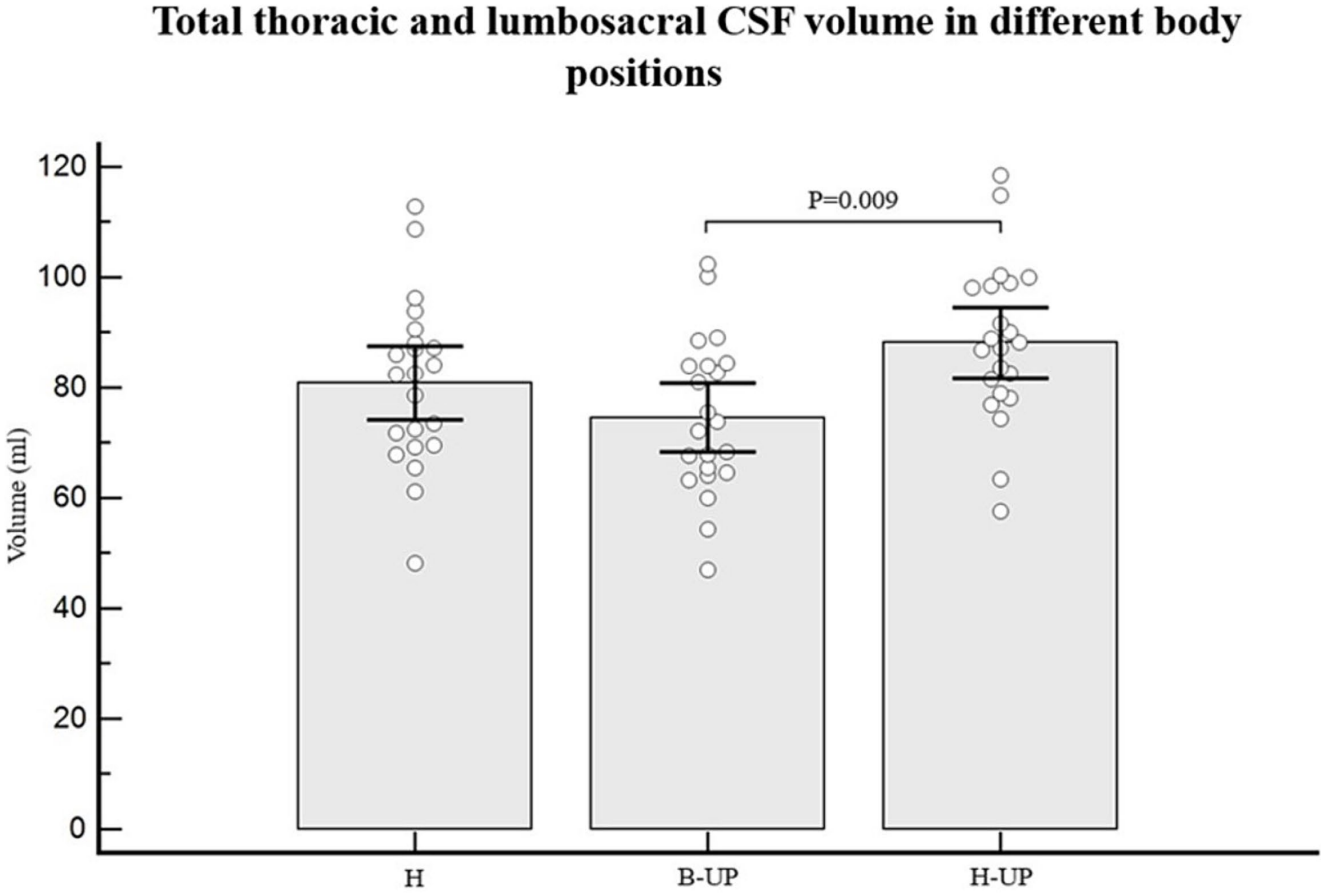
Differences between measured values of total thoracic and lumbosacral CSF volume (ml) related to the changes of body position during MRI imaging. Columns represent the values of arithmetic means of the total thoracic and lumbosacral CSF volumes with their 95% confidence intervals in horizontal position (H), in the position with elevated lower body (B-UP) and in the position with elevated head and upper body (H-UP), while white circles represent individual values of those volumes in 22 subjects. A post-hoc Bonferroni analysis showed significant differences primarily between the elevated head and upper body position (H-UP) and the elevated lower body position (B-UP) (*p* = 0.009).

As for individual data, if we look at the results regarding CSF volumes in the lumbosacral part, 19 out of 22 subjects had lower CSF volume in the B-UP position compared to the horizontal position, while 21 out of 22 subjects had higher CSF volume in the H-UP position compared to the horizontal position. The analysis of variance was used to study CSF volume values in the lumbosacral segment in 22 subjects and in three different body positions, and a statistically significant difference was obtained between different body positions on MRI imaging (*p* < 0.001) (Figure 5). An additional *post-hoc* Bonferroni analysis showed significant differences primarily between H position and B-UP position (*p* = 0.016), and between the H-UP and B-UP positions (*p* < 0.001). The highest average values were in the H-UP position (38.1 ± 7.0 mL), followed by the H position (34.4 ± 6.5 mL), while the lowest values were in the B-UP position (28.7 ± 6.5 mL). We also analyzed volumes of the total CSF in the thoracic and lumbosacral parts of the spine in 22 subjects and in three different body positions (Figure 6). The analysis of variance was performed and a significant difference was detected between three different body positions (*p* = 0.011) regarding the total thoracic and lumbosacral CSF volume. An additional *post-hoc* Bonferroni analysis showed a significant difference primarily between the H-UP and B-UP positions (*p* = 0.009). The highest average CSF volume values in the thoracic and lumbosacral parts were in the H-UP position (88.1 ± 14.5 mL), while they were lowest in the B-UP position (74.6 ± 14.1 mL). The average total thoracic and lumbosacral CSF volume in the H position measured 80.8 ± 15.1 mL.

### Influence of body position on the total craniospinal CSF volume distribution

The average total intracranial and spinal CSF volume measured 292.2 ± 55.4 mL in the H position and 286.8 ± 53.9 mL in the B-UP position, while average total intracranial and cervical CSF volume measured 211.4 ± 46.7 mL in the H position and 212.2 ± 45.2 mL in the B-UP position. A one-way analysis of variance showed no statistically significant difference between the mentioned volumes, which implies that body position changes had no significant influence on the CSF volume.

## Discussion

Our study shows that the amount of total craniospinal CSF volume in healthy volunteers is much higher than stated in the current textbooks, and that changes in body position lead to CSF volume alterations in line with the new hypothesis according to which spinal intradural space, due to its elasticity, can significantly change its volume, thus adjusting to the influence of gravity and other pathophysiological changes of neurofluid volume and pressure. Namely, significant CSF volume changes occur inside the spinal space in the tested body positions, primarily in the lumbosacral segment (Figures 5, 6), while at the same time no significant CSF volume changes have been observed inside the cranium in two tested positions in which it was possible to measure intracranial CSF volume changes (Figure 3), or if we added up cervical and cranial CSF volumes in those positions (see Results).

### Correlation between participant age/height and CSF volume

Most previously published studies showed that the total intracranial CSF volume is about 150 mL with 25 mL inside the lateral ventricles (Sakka et al., 2011; Naidich et al., 2013; Brinker et al., 2014; Miyajima and Arai, 2015). More recent studies reveal that intracranial volumes change linearly, depending on the participants’ age (Beheshti et al., 2019; Statsenko et al., 2021; Yamada et al., 2023). In the study done on 133 healthy volunteers between 21 and 92 years of age, an increase of intracranial CSF volume of about 30 mL per decade, starting from 265 mL in the twenties and up to 488 mL above 80 years was observed (Yamada et al., 2023). It was detected that CSF volume inside the lateral ventricles changes very little up to 60 years (average volume around 20 mL), however, it significantly increases after 60 years of age. The average age of our subjects of both genders was 25.5 years (20–34 years), and the total cranial CSF volume was about 184 mL with 13 mL inside the lateral ventricles, which fits well with the mentioned study results.

The total spinal CSF volume was about 81 mL (range 52–103 mL) in 22 healthy elderly volunteers, of which the cervical CSF volume was about 19 mL, the thoracic CSF volume was about 38 mL and the lumbosacral CSF volume was about 25 mL (Edsbagge et al., 2011). Spinal CSF volume in that age group did not significantly correlate with the subject gender or height. Contrary to that, a study done on pediatric population (Jang et al., 2019) showed linear correlations with the subject height and weight. The mean thoracolumbosacral CSF volume per weight (ml/kg) was 1.95 in neonates and infants, 1.82 mL in toddlers and preschoolers, 1.38 in schoolers and 0.99 in adolescents (Jang et al., 2019). In our study on younger healthy volunteers, the average value of the total spinal CSF volume was 108.8 mL, and it was observed that there is a correlation between their height and CSF volume in the spinal subarachnoid space (Table 1).

**Table 1:** Correlations between the subjects age and height, and their spinal, cranial and total spinal + cranial CSF volumes (volume values from the horizontal position) shows a significant positive correlation between the age and the cranial CSF volume (correlation coefficient = 0.432), as well as between the age and the total spinal + cranial CSF volume (correlation coefficient = 0.336).

Correlations					
			CSF spinal horizontal (ml)	CSF intracranial horizontal (ml)	Total CSF intracranial + spinal horizontal (ml)
Kendall tau_b coefficient	Age	Correlation coefficient	0.038	0.432**	0.336*
		*p*	0.815	0.009	0.041
		*N*	22	22	22
	Height (cm)	Correlation coefficient	0.329*	0.039	0.110
		*p*	0.034	0.799	0.480
		*N*	22	22	22

* Correlation is significant at the 0.05 level (2-tailed). ** Correlation is significant at the 0.01 level (2-tailed). The spinal CSF volume is significantly positively correlated to height (correlation coefficient = 0.329).

Thus, by analyzing the existing results from the literature as well as our results, it can be concluded that a significant correlation exists between the participants’ age and the intracranial CSF volume, as well as between their age and the total craniospinal CSF volume, which means that older age is related to a larger CSF volume both inside the cranium, and inside the cranial and spinal space combined. The spinal CSF volume in healthy young volunteers was also positively correlated to their height, which implies that higher participants have larger spinal CSF volumes. However, our previous study on NPH patients older than 65 did not show that the spinal CSF volume correlates significantly with the patients’ height (Kudelić et al., 2023) which is similar to results from another study on healthy elderly volunteers (Edsbagge et al., 2011). Thus, it seems that age and height differences between subjects can only partly explain the variations in results obtained by a volumetric analysis of the total intracranial CSF in mentioned studies.

Both in this study on healthy young volunteers as well as in our previous study on NPH patients in which total, cranial and spinal CSF volumes were determined, it can be easily observed that the total CSF volumes in certain individuals were significantly larger than it was previously published. Namely, in the NPH patients’ study, the total CSF volumes varied from 254.8 mL to 594.1 mL (mean 422.8 ± 108.4 mL) (Kudelić et al., 2023). Even when 70 mL (an average increase of the ventricular volume due to hydrocephalus) was subtracted, the total volume was still significantly larger (around 350 mL) than it was previously believed (around 150 mL). In this study on healthy volunteers, the total CSF volume varied from an average of 292.2 ± 55.4 mL in the horizontal position to 286.8 ± 53.9 mL in the B-UP position (see Results). It can be concluded from our two craniospinal volumetry studies that both total and cranial CSF volumes notably depend on the subject age and that they increase as one is getting older.

### Influence of body position changes on the intracranial CSF volume

Despite the fact that the craniospinal CSF system makes a sole functional unit, most investigations are limited to the analysis of the cranial CSF space, completely disregarding the spinal part of the CSF system. Our previous experimental observations on cats and on a model show that changes of body position can significantly expand or narrow the spinal dura in the lumbosacral and vertebral segments (Klarica et al., 2014, 2019), which is why our earlier publications (Klarica et al., 2009; Bulat and Klarica, 2011; Jurjević et al., 2011) described the spinal dural space as the dominant site for the compensation of acute volume changes inside the craniospinal CSF space. Volume changes in the lumbosacral segment have been noticed even before (Martins et al., 1972) during various physiological processes such as changes of breathing depth, Valsalva test, etc. Keeping in mind the biophysical characteristics of the dura (Tunturi, 1978), we assumed that body position changes, due to the influence of hydrostatic forces, will lead to the widening of certain parts of the CSF system (most prominently at the site with the highest hydrostatic force, i.e., the lumbosacral part) and to CSF redistribution predominantly inside the spinal CSF system which can alter its volume, while the intracranial CSF volume would remain within the normal range, without statistically significant variations.

In two described body positions (H and B-UP) there was no significant change of the intracranial CSF volume. Moreover, with further detailed analyses no significant CSF volume changes were observed in the subarachnoid space or in the ventricular system (Figure 3). A classical hypothesis assumes that during the head-up verticalization a significant CSF shift occurs into the spinal part of the CSF system under the influence of gravity (Magnæs, 1976a,b, 1989; Magnaes, 1978; Davson et al., 1987). It was observed with MRI and ultrasound that during body verticalization there is a simultaneous CSF shift in the cervical segment together with cervical vein expansion (Alperin et al., 2005a,b), which indirectly suggests that CSF moves from the cranial into the spinal compartment. Furthermore, it is believed that this CSF displacement is the cause of a short-term pressure decrease during the head-up verticalization. Namely, numerous studies have noted that during the changes of body position from the horizontal to sitting or standing positions there is a drop of intracranial pressure which often reaches subatmospheric values (Masserman, 1934; Loman, 1935; Von Storch, 1937; Bradley, 1970; Fox et al., 1973; Portnoy et al., 1973; McCullough and Fox, 1974; Chapman et al., 1990; Antes et al., 2016). According to the classical concept, this pressure change is short-lasting considering the constant CSF secretion, thus compensating the displaced volume. However, it was described on MRI imaging done with the changes of body position from the horizontal to head-up that the cervical section of CSF space becomes narrower (Alperin et al., 2005a,b). This data suggests that CSF is more significantly displaced from the cervical segment into the hydrostatically lower parts than it is shifted from the cranial into the spinal part, as the latter would lead to the widening of the cervical segment, or it should at least stay the same as before the changes of body position.

Our results cannot be explained by the classical hypothesis, however, they fit into the new concept of CSF physiology created by our research group, with one of the basic postulates being the preservation of neurofluid volumes (blood, CSF, interstitial fluid) inside the intracranial space (Orešković et al., 2002, 2017a,b; Orešković and Klarica, 2010, 2011, 2014; Bulat and Klarica, 2011; Klarica et al., 2014; Radoš et al., 2014; Klarica et al., 2019). Namely, that concept interprets the mentioned CSF pressure decrease by the physical laws that can be applied to the fluids enclosed inside the spaces with hard walls (spaces with low elasticity) which cannot significantly alter their volumes (Klarica et al., 2014). Our hypothesis presumes that the CSF volume inside the intracranial space is only modestly changeable, and that it is not significantly dependent on the body position. Thus, during the changes of body position, the CSF pressure inside the cranial space changes significantly, however, without significant CSF volume changes (Klarica et al., 2014).

It appears that the cranial space preserves volume, since it was also noticed that there is no significant intracranial CSF volume change during the changes of its volume throughout the entire craniospinal space due to the lumbar CSF extraction (Alperin et al., 2016; Nikić et al., 2016). Thus, the CSF volume decrease in the spinal part caused by lumbar drainage did not lead to significant CSF volume changes inside the cranium, i.e., there was no extraction or redistribution of CSF from the cranial into the spinal part, even though the CSF pressure certainly changed significantly.

### Distribution of the spinal CSF volume during the changes of body position

The total spinal CSF volume was without any significant change (Figure 4), which implies that there was no notable CSF displacement from the cranial into the spinal part during body position changes (H to B-UP) as it was previously assumed, but there was primarily a redistribution of CSF volume within the spinal part of the system. The spinal CSF redistribution is best displayed in Figure 5, which shows the results of a lumbosacral segment volumetric analysis, thus confirming our expectation that CSF redistribution will be most pronounced in the most caudal part of the spinal system, in which epidural space is the widest and where hydrostatic pressure is predominantly changed.

During the change of body position from H to B-UP there is a statistically significant CSF volume reduction in the lumbosacral segment (*p* < 0.016). An additional volumetric analysis shows that the lumbosacral CSF volume is significantly larger in the H-UP position compared to the previous B-UP position (*p* < 0.001). Results imply that the dural sac in the lumbosacral segment can significantly alter its volume (the smallest average CSF volume in the lumbosacral segment was 28.7 mL in the B-UP position, while the largest volume was 38.1 mL in the H-UP position), depending on the fullness of the CSF system. Thus, it seems that lifting the head and upper body leads to CSF redistribution from the cervical and thoracic segment into the more caudal lumbosacral segment under the influence of gravity. This redistribution is biophysically possible due to the fact that spinal dura can be significantly narrowed and distended since it is not firmly attached to the bone as it is in the cranium, but it hangs freely inside the spinal canal (Martins et al., 1972).

With lifting the lower part of the body, our results show a decrease of CSF volume inside the lumbosacral segment, probably due to the CSF redistribution to the more cranial parts of the spinal CSF system. In this position we also measured the intracranial CSF volume which did not differ significantly compared to the horizontal position, so we can conclude that the CSF shift is predominantly restricted to the spinal part of the CSF system.

From a biophysical standpoint, a slight additional volume shift is possible from the cranium into the perioptic space (in the B-UP position), as well as from the perioptic space into the cranium and from the cranium into the spinal space (in the H-UP position). Since the CSF volume surrounding the optical nerve is very small, that shift should not lead to any significant change of the spinal volume (as can be seen in Figure 4). As a limitation of this study, it should certainly be pointed out that we observed CSF volume changes during the changes of the body position mostly 30 degrees from the position of the head. It is possible that more pronounced changes in the vertical head-up body position (90 degrees) or vertical head-down position (270 degrees) would also lead to more significant CSF volume redistribution inside the spinal canal.

### Clinical implications of our results

The results obtained in this research cannot be explained by a classical concept of CSF physiology, but they fit into the new concept designed by our research group, according to which biophysical characteristics of the craniospinal system are of utmost importance for the understanding of the changes of CSF pressure and volume during the changes of body position.

It is our belief that the negative intracranial pressure during the head-up verticalization is not a consequence of significant CSF shift, however, it can be explained by the Law of fluid mechanics which describe how fluid (CSF) acts inside the rigid (cranial) space opened at the bottom (foramen magnum), while the spinal part is pivotal for the compensation of volume changes throughout the entire craniospinal CSF system due to its unique biophysical characteristics (Klarica et al., 2014). The cranium plays an important role in the prevention of significant changes in the volumes of blood, CSF and brain parenchyma, and it does not allow any sudden changes of those volumes during normal daily activities, enabling an adequate brain perfusion in the vertical head-up position. This can be corroborated by numerous clinical issues that patients often have after craniectomy (Ashayeri et al., 2016; Tarr et al., 2020; Mustroph et al., 2022).

The mentioned redistribution of CSF inside the spinal canal due to the changes of body position could provide a reason for faster redistribution of the substances applied into the cisterna magna within the spinal subarachnoid space compared to their distribution into the cranial space if the human subjects or experimental animals move freely post application (Vladić et al., 2000, 2009; Klarica et al., 2019). Namely, CSF volume movement, which during inactivity mostly occurs due to pulsations (Orešković and Klarica, 2014), is additionally enhanced during body position changes due to gravitational redistribution inside the spinal canal (Klarica et al., 2019). This phenomenon significantly affects the distribution of both metabolites and drugs applied intrathecally for various indications (Kouzehgarani et al., 2021).

## Conclusion

Changes of body position from horizontal to those with head above or below level of the remaining body caused significant CSF volume redistribution inside the spinal subarachnoid space of healthy volunteers, while CSF volume inside the cranium did not significantly change, nor did the volumes of brain tissue and spinal cord.

## Limitations

In this research, we had a relatively small number of subjects, although sufficient for statistical analysis, with whom we changed the body position by only 30 degrees. Body position changes are significantly limited by the width of the tunnel (60 cm) in which the patient is located during MR imaging. Another important limitation concerns the coils, which must be placed directly next to the part of the body being recorded. Due to the mentioned technical limitations, intracranial CSF volumes can only be recorded in the horizontal position (H) and in the position with raised lower part of the trunk (B-UP). It would be very meaningful to record intracranial and spinal volumes in the head-up (H-UP) position, as we would expect even more significant changes in terms of cervico-lumbar redistribution of the CSF volume.

## Data Availability

The raw data supporting the conclusions of this article will be made available by the authors, without undue reservation.
