# Supplementary material for: Body position influence on cerebrospinal fluid volume redistribution inside the cranial and spinal CSF compartments
Source: Front Hum Neurosci. 2025 Jan 29;18:1463740. doi: 10.3389/fnhum.2024.1463740 (PMC11814182; doi:10.3389/fnhum.2024.1463740)
Supplement: Supplementary file 1 [file Data_Sheet_1.PDF]

Table S1. Gender (F- female gender; M – male gender), age (years), height (m), weight (kg) and body mass index (BMI) of each subject.

SUBJECT	GENDER	AGE (years)	HEIGHT (m)	WEIGHT (kg)	BMI
**1**	F	31	1.58	50	20.0
**2**	F	20	1.63	53	19.9
**3**	F	20	1.68	62	22.0
**4**	F	21	1.65	56	20.6
**5**	M	34	1.77	73	23.3
**6**	F	31	1.70	70	24.2
**7**	F	31	1.58	60	24.0
**8**	M	31	1.75	87	28.4
**9**	M	26	1.85	110	32.1
**10**	M	33	1.87	87	24.9
**11**	F	31	1.73	70	23.4
**12**	M	30	1.85	95	27.8
**13**	M	31	1.83	95	28.4
**14**	M	21	1.82	92	27.8
**15**	M	26	1.83	90	26.9
**16**	M	20	1.79	75	23.4
**17**	F	20	1.65	55	20.2
**18**	M	21	1.84	80	23.6
**19**	M	21	1.75	75	24.5
**20**	F	21	1.70	55	19.0
**21**	F	21	1.67	67	24.0
**22**	F	21	1.80	77	23.8
